# Improved efficacy of mesenchymal stromal cells stably expressing CXCR4 and IL-10 in a xenogeneic graft versus host disease mouse model

**DOI:** 10.3389/fimmu.2023.1062086

**Published:** 2023-02-01

**Authors:** Rosario Hervás-Salcedo, María Fernández-García, Miriam Hernando-Rodríguez, Cristian Suárez-Cabrera, Juan A. Bueren, Rosa M. Yáñez

**Affiliations:** ^1^ Hematopoietic Innovative Therapies Division, Centro de Investigaciones Energéticas, Medioambientales y Tecnológicas (CIEMAT) and Centro de Investigación Biomédica en Red de Enfermedades Raras (CIBERER), Madrid, Spain; ^2^ Advanced Therapies Unit, Centro de Investigaciones Energéticas, Medioambientales y Tecnológicas (CIEMAT)/Instituto de Investigación Sanitaria (IIS) Fundación Jiménez Díaz, Madrid, Spain; ^3^ Translational Oncology Division, Centro de Investigaciones Energéticas, Medioambientales y Tecnológicas (CIEMAT) and Centro de Investigación Biomédica en Red de 8 Cancer (CIBERONC), Madrid, Spain; ^4^ Biomedical Research Institute I + 12, Hospital 12 de Octubre, Madrid, Spain

**Keywords:** mesenchymal stromal cells, graft vs host disease, CXCR4, IL10, stem cell therapy, immunomodulation, lentiviral vector

## Abstract

Previous clinical trials have shown that mesenchymal stromal cells (MSCs) can modulate graft versus host disease (GvHD) after allogeneic hematopoietic transplantation, although with variable efficacy. To improve the anti-GvHD effect of these cells, adipose tissue derived-human MSCs (Ad-MSCs) were transduced with a lentiviral vector conferring stable expression of CXCR4, a molecule involved in cell migration to inflamed sites, and IL-10, a cytokine with potent anti-inflammatory properties. *In vitro* experiments showed that the expression of these molecules in Ad-MSCs (named CXCR4-IL10-MSCs) efficiently enhanced their migration towards SDF-1α and also improved their immunomodulatory properties compared to unmodified Ad-MSCs (WT-MSCs). Moreover, using a humanized GvHD mouse model, CXCR4-IL10-MSCs showed improved therapeutic effects, which were confirmed by histopathologic analysis in the target organs. Additionally, compared to WT-MSCs, CXCR4-IL10-MSCs induced a more marked reduction in the number of pro-inflammatory Th1 and Th17 cells, a higher polarization towards an anti-inflammatory T cell profile (CD3^+^-IL10^+^ cells), and increased the number of regulatory T and B cells. Our *in vitro* and *in vivo* studies strongly suggest that CXCR4-IL10-MSCs should constitute an important new generation of MSCs for the treatment of GvHD in patients transplanted with allogeneic hematopoietic grafts.

## 1 Introduction

Allogeneic hematopoietic stem cell transplantation (alloHSCT) is the treatment of choice for numerous hematological malignancies (1, 2). Nevertheless, one of the major complications associated with alloHSCT is acute graft versus host disease (GvHD) (3), which occurs in approximately 50% of patients transplanted with alloHSCs. Since acute GvHD is mainly mediated by effector T cells, prophylactic strategies with immunosuppressive agents are used to prevent GvHD (4). Despite these prevention strategies, 30% to 60% of transplanted patients require systemic glucocorticoid therapy as first line of treatment. Unfortunately, around half of these patients become refractory to steroids (SR-GvHD), and thus have a serious risk of mortality (5). While ruxolitinib, a specific inhibitor of JAK1/2 kinases, has been recently proposed as a second line of treatment (6), cell therapy with mesenchymal stromal cells (MSCs) constitutes an additional therapy frequently used for the treatment of GvHD.

Based on the immunomodulatory properties of MSCs in both the innate (7–10) and adaptive (11–13) immune system, in 2004 Le Blanc and coworkers treated for the first time a pediatric patient with severe SR-GvHD with MSCs (14). Five years later, the first phase III clinical trial with allogeneic MSCs was carried out for the treatment of SR-GvHD (15). Subsequently, two MSC medicinal products were approved in Canada and Japan for the treatment of specific patients with SR-GVHD. Nevertheless, the variable response rates observed in different MSC-based clinical trials highlight the need for improving the efficacy of these cells in patients with GvHD (16–20).

Advances in the field of MSC-based therapies aim at improving the therapeutic efficacy of these cells using innovative approaches which include the genetic modification of these cells to enhance their ability to migrate towards inflamed sites, and also to potentiate their immunomodulatory properties (19). Previous studies have shown that the expression of CXCR4 in MSCs enhanced their migration towards inflamed tissues in different animal models (21–23). Additionally, anti-inflammatory cytokines, such as IL-10, have been ectopically expressed in MSCs to improve their immunomodulatory properties (24–26).

In a previous study, we showed in an acute local inflammation model that the mRNA-mediated transient co-expression of CXCR4 and IL-10 confers enhanced anti-inflammatory properties to adipose tissue-derived MSCs (Ad-MSCs), compared to the effects mediated by each of these molecules in these cells (27). More recently, the genetic modification of enucleated MSCs with constructs promoting the expression of IL-10 and different homing receptors, which included CXCR4, also improved the therapeutic efficacy of WT-MSCs in a different local inflammation model, as well as in a disease model of acute pancreatitis (28).

In the current study, we demonstrate that, compared to WT-MSCs, MSCs stably expressing both CXCR4 and IL-10 exert enhanced therapeutic effects in a humanized model of GvHD, strongly suggesting that these cells will constitute a novel advanced therapy medicinal product with enhanced anti-GvHD therapeutic properties.

## 2 Materials and methods

### 2.1 Generation and expansion of adipose derived-MSCs

Adipose tissue samples were purchased from Caltag MedSystem (Buckingham, UK). The project was approved by the Ethics Committee of Hospital Fundación Jiménez Díaz (Madrid, Spain). Adipose tissue was disaggregated and digested with collagenase A (Serva, Heidelberg, Germany) at a final concentration of 2 mg/ml for 4 h at 37°C. Digested samples were filtered through 100-μm nylon filters (BD Bioscience, NJ, USA) and centrifuged for 10 min. The cell pellet was re-suspended in Minimum Essential Medium α (ɑ-MEM; Gibco/Life Technologies/Thermo Fisher Scientific, Waltham, USA) supplemented with 5% platelet lysate (Cook Medical, IN, USA), 1% penicillin/streptomycin (Gibco/Life Technologies/Thermo Fisher Scientific, Waltham, USA), and 1 ng/ml human basic fibroblast growth factor (bFGF; Peprotech, NJ, USA). Cells were seeded at a concentration of 10,000 cells/cm^2^ in culture flasks (Corning, NY, USA) and cultured at 37°C. For the expansion of adipose tissue derived-MSCs (Ad-MSCs), the cell medium was changed every 2–4 days and adherent cells were serially passaged using 0.25% trypsin/EDTA (Sigma Aldrich, St. Louis, MO, USA) upon reaching near confluence (70%–90%).

### 2.2 Lentiviral vector production

Codon-optimized sequences of human *CXCR4* and *IL10* cDNAs (GeneScript, NJ, USA) were cloned into a pCCL plasmid as a bicistronic construct using the E2A sequence to facilitate the co-expression of both proteins under the human *phosphoglycerate kinase promoter* (PGK). pCCL backbone was obtained from the pCCL.PGK.GFP.WPRE LV kindly provided by Luigi Naldini (Istituto Scientifico H San Raffaele. Milano. LV containing supernatants were produced by transient co-transfection of HEK-293T cells in the presence of CaCl_2_ 2.5M using equimolecular mixtures of the envelope (pMD2.VSVg) and packaging (pCMVdR8.74) plasmids produced by Plasmid Factory (Bielefeld, Germany). The lentiviral supernatants were concentrated by ultracentrifugation at 20,000 r.p.m. for 2 hours at 10°C. The viral titer was determined by transducing HEK-293T cells with serial dilutions of the LV.

### 2.3 Lentiviral transduction of Ad-MSCs

Ad-MSCs (passages 1 or 2) were transduced in suspension using a multiplicity of infection (MOI) of 50 infective LVs/cell. Cells were trypsinized and cell pellets were resuspended with the corresponding volume of LV supernatant and incubated for 10 min at room temperature using poloxamer F108 (Sigma-Aldrich) as transduction enhancer. Cell suspensions were then centrifuged at 800xg for 30 min and reseeded. Next day, supernatants of transduced Ad-MSCs (named as CXCR4-IL10-MSCs) were replaced with for fresh complete ɑ-MEM medium. For *in vitro* and *in vivo* studies, CXCR4-IL10-MSCs and the corresponding WT-MSC controls were used at passages 4 to 8.

### 2.4 Vector copy number analysis

After 2 weeks of *in vitro* culture, CXCR4-IL10-MSCs were collected for genomic DNA (gDNA) extraction and subsequent analysis of the integrated vector copy number per cell (VCN/cell).The gDNA was amplified by quantitative polymerase chain reaction (qPCR) using the TaqMan mixture (Thermo Fisher Scientific, USA) with specific primers and probes for *Ps*i sequence and human albumin gene (**Table S1**) in a 7500 Fast Real-Time PCR System (AppliedBiosystems, USA). The vector copy number per cell was analyzed by duplex detection of *Psi* packaging sequence normalized to human albumin.

### 2.5 Characterization of CXCR4-IL10-MSCs

#### 2.5.1 Immunophenotype

WT and CXCR4-IL10-MSCs were immunophenotypically characterized by flow cytometry (Fortessa, BD Bioscience, NJ, USA) as described by the Mesenchymal cell kit (Immunostep, Salamanca, Spain). The monoclonal anti-human antibodies included in these studies were the following: CD29, CD44, CD73, CD90, CD105, CD166, CD45, CD19, HLA-DR, CD14, and CD34. Data were analyzed with FlowJo version X (FlowJo LLC, CA, USA).

#### 2.5.2 *In vitro* differentiation capacity

The osteogenic and adipogenic differentiation ability of WT and CXCR4-IL10-MSCs was determined using the NH-OsteoDiff and NH-AdipoDiff Media (Miltenyi Biotec, Bergisch Gladbach, Germany), respectively, according to the manufacturer’s protocols. Alkaline phosphatase deposits were observed after the staining with Fast BCIP/NCP (Sigma Aldrich, St. Louis, MO, USA) while lipid droplets were seen with optic microscopy (Nikon, Düsseldorf, Germany). Gene expression analyses related to the differentiation to bone and adipose tissue was performed after 10 or 21 days of culture, respectively, by qRT-PCR, following RNA extraction with RNeasy^®^ Plus Mini Kit (QIAGEN) and reverse transcription with RETROscript kit (Thermo Fisher Scientific). qRT-PCR was performed using TaqMan™ Fast Advanced Master Mix (Thermo Fisher Scientific) and mixes of probes and primers specific for *BGLAP* (Ref: Hs01587814_g1), *ALPL* (Ref: Hs01029144_m1) and *PPARγ* (Ref: Hs01115513_m1), all conjugated with FAM (ThermoFisher Scientific), on QuantStudio™ 6 Flex Real-Time PCR System (Applied Biosystems). Results were normalized for human *GAPDH* expression (Ref: Hs02786624_g1, conjugated with VIC; Thermo Fisher Scientific) and the expression of control samples according to the 2^-ΔΔCt^ method.

### 2.6 Secreted factors and cytokines quantification

Fifteen days after transduction, supernatants from WT-MSCs and CXCR4-IL10-MSCs were replaced by fresh medium, which was collected 24 hours after changing. Cytokines and factors secretion was quantified by flow cytometry using the LEGENDplex™ Multi-Analyte Flow Assay Kit: Human Th Cytokine Panel and the Human Essential Immune Response Panel (Biolegend, USA). These data were analyzed with the kit’s own data analysis program. Levels of secreted PGE_2_ and TGFβ1 were quantified by ELISA (R&D System, USA).

### 2.7 Cell migration assay

Migration assays were carried out in transwells with an 8-μm pore polycarbonate membrane insert (Costar, Cambridge, MA) and 5×10^3^ WT-MSCs or CXCR4-IL10-MSCs were placed in the upper insert chamber of the transwell assembly. The lower chamber contained murine or human SDF-1 (Peprotech, NJ, USA) at a final concentration of 100 ng/ml. Twenty-four hours after incubation, the upper part of the membrane was scrapped gently by a cotton swab to remove non-migrating cells and washed with PBS. Membranes were then fixed with 3.7% formalin overnight at 4°C and stained with hematoxylin for 4h at RT. The number of migrating cells was determined by the scoring of four random fields/well under the Nikon Eclipse E400 microscope (10X) (Nikon, Greater London, UK), and pictures were obtained with a Leica DFC420 camera (Leica, Buckinghamshire, UK).

### 2.8 *In vitro* immunosuppression assay

Heparinized peripheral blood samples from healthy donors were obtained from the Madrid Community Transfusion Centre according to their Institutional Review Board (IRB) approval and written informed consents in compliance with the Helsinki Declaration. MNCs were obtained by Ficoll-Paque PLUS (GE Healthcare Bioscience, Uppsala, Sweden) density gradient from heparinized PB samples. MNCs were stained with the intracellular fluorescent dye Carboxyfluorescein diacetate succinimidyl ester (CellTrace™ CFSE Cell Proliferation Kit; Molecular Probe/Invitrogen, USA), following a previously described protocol^18^. Before co-culture, WT and CXCR4-IL10-MSCs were plated in 24-well plates at a concentration of 5×10^4^ cells/well. Twenty-four hours later, 5×10^5^ stained MNCs were added to each well in the presence of 200 ng/ml of monoclonal antibody anti-human CD3 (Clone OKT3; Biolegend) and 150 U/ml of human IL-2 (Peprotech) to induce the specific proliferation of T lymphocytes, in RPMI medium with 10% Hyclone serum and 1% penicillin/streptomycin. After 3 days of incubation, cells harvested from culture wells were analyzed by flow cytometry for cell proliferation. Data were analyzed with ModFit LT™ (Verity Software House, Topsham, ME, USA). MNCs were also collected to analyze the expression of transcription factors involved in T cell polarization (*T-BET, STAT6, c-MAF* and *FOXP3*), using human *GAPDH* as a housekeeper (**Table S2**). Total RNA was extracted and cDNA was synthesized, as described previously. In addition, supernatants were collected to quantify cytokines and soluble factors related to T cell polarization. These factors were quantified by flow cytometry using the LEGENDplex™ Multi-Analyte Flow Assay Kit panel: Human Th1/Th2 Panel 8-Plex (Biolegend) and ELISA for IFNγ, TGFβ and IL-10 (Biolegend), following the manufacturer’s instructions for cell supernatant analysis.

### 2.9 Humanized graft-versus-host disease mouse model

Female NOD.Cg-PrkdscidIL2rgtm1Wjl/SzJ (NSG) mice (10–12 weeks old) were obtained from the Jackson Laboratory (Bar Harbor, ME) and were kept under standard pathogen-free conditions and given autoclaved food and water *ad libitum* in the animal facility of CIEMAT (Registration No. ES280790000183). All animal experiments were performed in compliance with the European and Spanish legislations and institutional guidelines (Spanish RD 53/2013, Law 6/2013, and European Directive 2010/63/UE). The protocol was approved by the CIEMAT Animal Experimentation Ethical Committee according to the approved biosafety and bioethics guidelines (Protocol number: PROEX 252-19). Mice were irradiated at 2.0 Gy using a MG324 X-ray irradiator (Philips, Germany) and injected *via* the tail vein with 5×10^6^ fresh human PB MNCs from healthy donor buffy coats 24 hours after irradiation. After 3 days, 1×10^6^ human unmodified WT-MSCs or CXCR4-IL10-MSCs were suspended in 300 µl of PBS and injected *via* the tail vein. Clinical signs of GvHD were evaluated daily by examining body weight loss, diarrhea, hunched back, activity, fur texture and skin integrity, and were recorded every second day following scoring criteria in **Table S2**. Mice were sacrificed three weeks after transplantation and a thorough analysis of human chimerism was carried out in different organs.

### 2.10 Human chimerism analysis

#### 2.10.1 Flow cytometry

Cells from recipients’ PB and spleen were stained with antibodies described in **Tables S3** and **S4**. For intracellular analysis of cytokine expression, spleen cell suspensions were stimulated with 1X Cell stimulation Cocktail plus protein transport inhibitors (Thermo Fisher Scientific) for 12-14h. Cells were fixed and intracellularly stained with antibodies described in **Table S5** following BD Pharmingen™ Transcription Factor Buffer Set (BD Biosciences) instructions. Precision Count Beads (Biolegend) were added to peripheral blood and spleen prior to acquisition and the absolute numbers were calculated following the manufacturer’s instructions.

#### 2.10.2 Circulating cytokine analysis

Mouse PB samples were centrifuged to separate the serum. A total of 13 cytokines were analyzed by flow cytometry using LEGENDplex™ Human Inflammation Panel 1 (13-plex) (BioLegend). IFNγ, IL-10 and TGFβ levels were analyzed by ELISA (BioLegend), following the manufacturer’s instructions for the serum analysis.

### 2.11 Gene expression analysis

Cells obtained from the spleen of recipient mice were sorted using BD Influx™ Cell Sorter (BD Biosciences), in order to obtain a pure population of hCD45^+^ cells. Total RNA was extracted using the RNeasy^®^ Plus Mini Kit (QIAGEN) and quantified using the spectrophotometer NanoDropTM One (ThermoFisher Scientific). Its integrity was analyzed using the Qubit™ RNA IQ kit Assay and the Qubit™ 4 fluorimeter (ThermoFisher Scientific). Messenger RNA sequencing and bioinformatic data processing were performed by Diagenode (Belgium), following expert staff criteria. Comparisons of the gene expression of immune signatures were performed using the GSEA program (Gene Set Enrichment Analysis; USA) (29) using immunologic signature gene sets. Data from RNA-seq and GSEA analysis was validated with RT-qPCR. Total RNA from hCD45^+^ cells isolated from the spleen of recipient mice was subjected to reverse transcription with SuperScript™ VILO™ Master Mix (ThermoFisher Scientific). The cDNA was analyzed in a QuantStudio™ 6 Flex Real-Time PCR System (Applied Biosystems) using TaqMan™ Fast Advanced Master Mix (Thermo Fisher Scientific) and mixes of specific probes and primers for *IFNγ* (Ref: Hs00989291_m1), *IL17A* (Ref: Hs00174383_m1), *IL22* (Ref: Hs01574154_m1), *FoxP3* (Ref: Hs01085834_m1), *IL5* (Ref: Hs00174200_m1) and *IL10* (Hs00961622_m1), all conjugated with FAM (ThermoFisher Scientific). Results were normalized for the expression of human *GAPDH* (Ref: Hs02786624_g1, conjugated with VIC; Thermo Fisher Scientific) and the expression of control samples according to the 2^-ΔΔCt^ method.

### 2.12 Histology and immunohistochemistry

Mouse back skin, the small intestine, lungs and liver were dissected and immediately fixed in 10% buffered formalin and embedded in paraffin. Sections with a thickness of 3-5 µm were used for hematoxylin and eosin (H&E) staining or immunohistochemical preparations. Primary and biotinylated secondary antibodies used are listed in **Table S6**. Immunoreactivity was revealed using VECTASTAIN^®^ Elite ABC-HRP Kit and DAB Substrate Kit (Vector Laboratories; Burlingame, CA, USA), and the sections were counterstained with hematoxylin. The control experiments without the primary antibody gave no signals.

### 2.13 Statistical analysis

Statistical analyzes were performed using Graph Pad Prism 9.0 software (Graph Pad Software, USA). *In vitro* test data are expressed as mean ± standard deviation (SD) and as mean ± standard error of the mean (SEM) the *in vivo* tests. Normal distribution was analyzed using Shapiro-Wilks’ test. To compare more than two groups, parametric one-way ANOVA test was used for normal distribution, with subsequent Tukey’s *post hoc* analysis for multiple comparisons. When the distribution was not normal, non-parametric Kruskal-Wallis’ test was performed with Dunn’s *post hoc* analysis for multiple comparisons. In this study, p-values < 0.05 were considered statistically significant: * p < 0.05; ** p < 0.01; *** p < 0.001; **** p < 0.0001.

## 3 Results

### 3.1 Human Ad-MSCs transduced with a bicistronic CXCR4-IL10 lentiviral vector efficiently express both transgenes and maintain the characteristic phenotype of human MSCs

**Figure 1:**
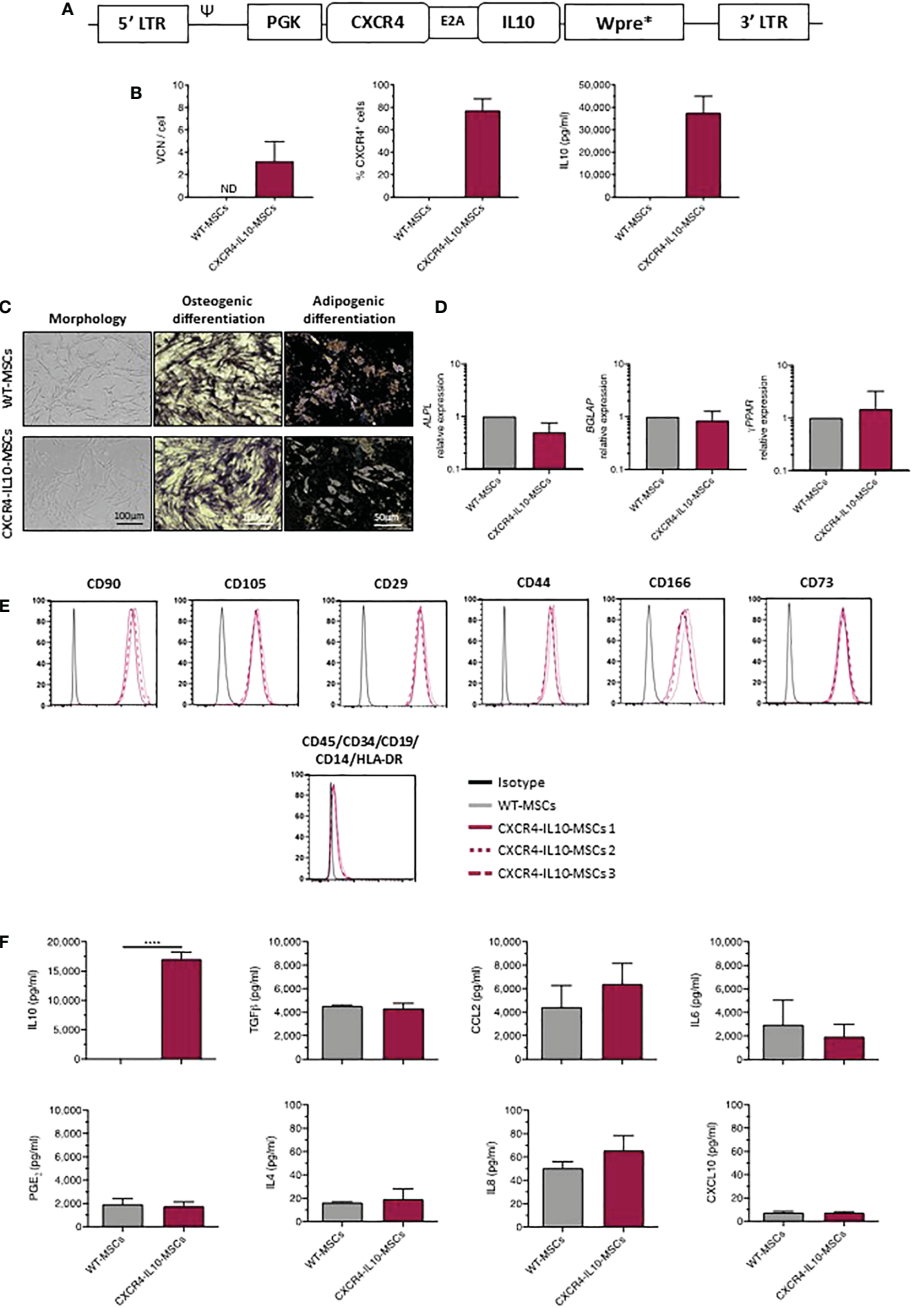
Phenotypic characterization of MSCs transduced with a lentiviral vector carrying the *CXCR4* and *IL10* cDNAs (CXCR4-IL10-MSCs). **(A)** Description of the bicistronic LV carrying the codon-optimized cDNA sequences of *CXCR4* and *IL10* (PGK.CXCR4-IL10.Wpre*) used to transduce MSCs. **(B)** Analysis of the vector copy number per cell (VCN/cell) and of the percentage of CXCR4^+^ MSCs (flow cytometry analysis) and levels of secreted IL-10 (ELISA analysis) corresponding to WT-MSCs and CXCR4-IL10-MSCs. Analyses were performed in cells maintained for 2 weeks in culture after transduction. **(C)** Representative images of morphology, osteogenic (alkaline phosphatase deposits) and adipogeneic differentiation (presence of lipid droplets) of WT-MSCs and CXCR4-IL10-MSCs **(D)** mRNA expression analyses showing *ALPL* and *BGLAP* expression, characteristic of osteogenic differentiated cells, and of *γ-PPAR*, characteristic of adipogeneic differentiated cells. **(E)** Immunophenotype analysis of undifferentiated WT-MSCs and CXCR4-IL10-MSCs. Representative analyses of characteristic MSC markers are shown. **(F)** Levels of secreted cytokines corresponding to WT-MSCs and CXCR4-IL10-MSCs determined 24h after medium change. Bars represent the mean ± SD of n = 3 different experiments. ****p<0.0001; ND = not detectable.

In this study, human Ad-MSCs (WT-MSCs) were transduced with a bicistronic lentiviral vector (LV) which carries the optimized codon sequences of human *CXCR4* and *IL10* cDNAs (LV PGK.*CXCR4-IL10*.Wpre*; **Figure 1A**). Three lines of genetically modified CXCR4-IL10-MSCs were generated, which harbored a mean vector copy number (VCN) of 3.22 ± 1.72 per cell. On average, 80 ± 10.48% of these cells expressed CXCR4 on their membrane and secreted high levels of IL-10, which contrasted with the non-detectable expression of these molecules in WT-MSCs (**Figure 1B**).

Next, we investigated if CXCR4-IL10-MSCs maintained the characteristics of WT-MSCs, as defined by the International Society for Cell Therapy (30). As shown in **Figure 1C**, CXCR4-IL10-MSCs showed the typical fibroblastoid morphology of WT-MSCs and capacity to differentiate towards osteogenic and adipogenic lineages, confirmed by the expression of differentiation-related genes analyzed by qPCR (**Figure 1D**). CXCR4-IL10-MSCs also expressed the characteristic immunophenotypic markers of WT-MSCs (**Figure 1E**). Additionally, CXCR4-IL10-MSCs secreted a similar cytokine profile than WT-MSCs, except for IL-10, which was exclusively detected in CXCR4-IL10-MSCs (**Figure 1F**).

### 3.2 CXCR4-IL10-MSCs show a preferential migration towards SDF-1 and exert enhanced immunosuppression in *in vitro* cultures

**Figure 2:**
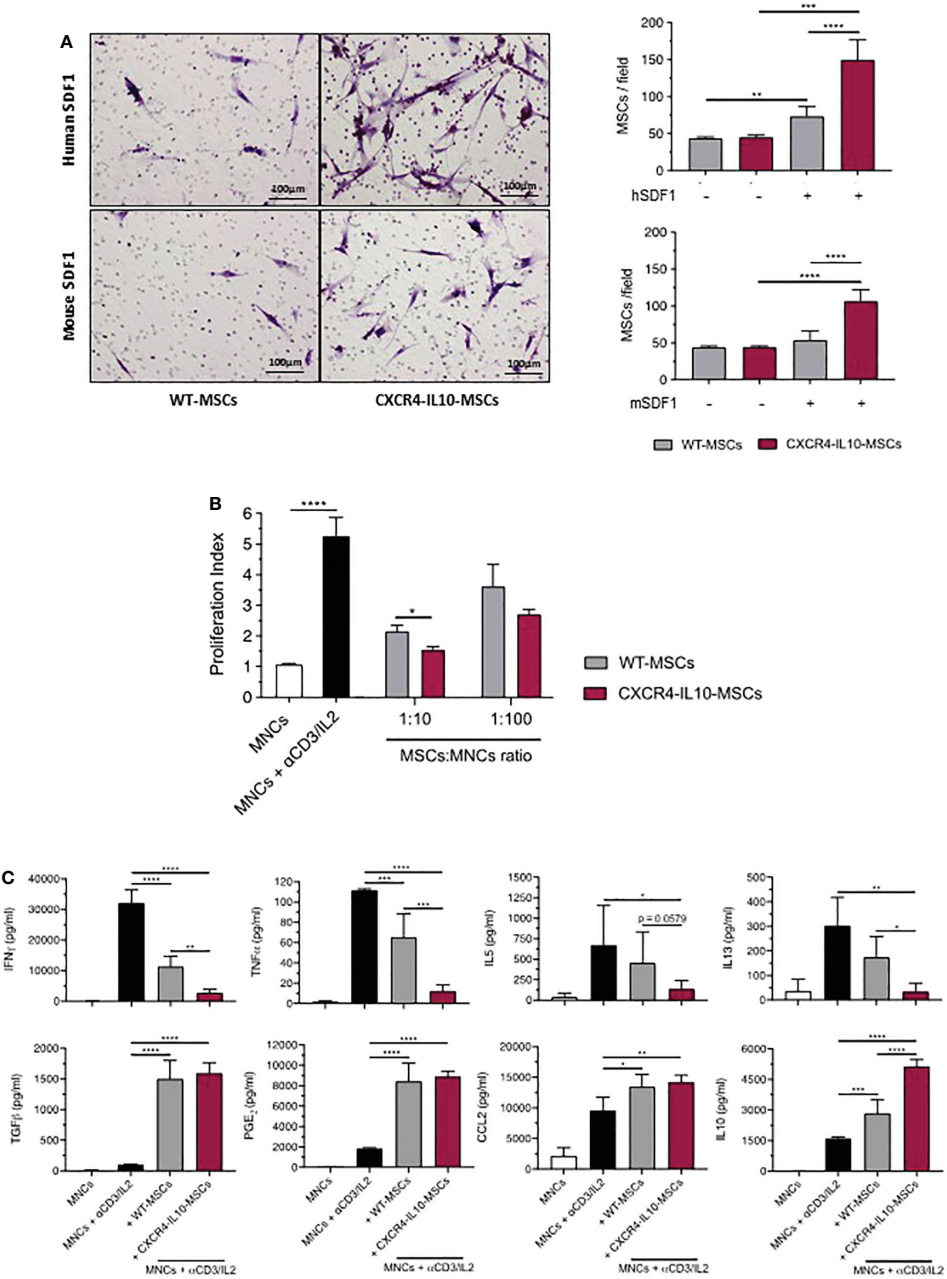
Enhanced migration towards SDF-1α and immunosuppression potential of CXCR4-IL10-MSCs. **(A)** Representative images of transwell-migration of WT-MSCs and CXCR4-IL10-MSCs in response to human and mouse SDF-1α. Cells in the transwell membranes were stained with hematoxylin. Right panels represent the mean number of MSCs per field that migrated in response to human and mouse SDF-1α. **(B)** *In vitro* immunosuppressive capacity of CXCR4-IL10-MSCs compared to WT-MSCs. The results correspond to the proliferative index of human T cells activated with anti-CD3/IL2 and treated with the corresponding Ad-MSCs. **(C)** Analysis of cytokine levels in supernatants collected 3 days after initiation of co-cultures (1:10 cell ratio). Bars represent the mean ± SD of n = 3-5 different experiments. *p < 0.05; **p < 0.01; ***p < 0.001. **** p <0.0001.

To study the functional properties of CXCR4-IL10-MSCs, a transwell migration assay in response to human and mouse SDF-1α was first performed. Compared to WT-MSCs, CXCR4-IL10-MSCs showed enhanced migration towards either human or mouse SDF-1α (**Figure 2A**). The ability of WT and CXCR4-IL10-MSCs to inhibit the proliferation of human T cells was comparatively evaluated in *in vitro* experiments. In these studies, CFSE-labelled human mononuclear cells (hMNCs) were incubated with anti-CD3 and IL-2 and co-cultured with WT and CXCR4-IL10-MSCs. Flow cytometry analyses performed three days after *in vitro* incubation showed that WT-MSCs inhibited T cell proliferation. Nevertheless, a higher inhibition was observed when CXCR4-IL10-MSCs were used (**Figure 2B** **)**. The secretion profile of culture supernatants showed that while WT-MSCs reduced levels of inflammatory cytokines such as IFNγ, TNFα and also of B cell activating cytokines (IL-5 and IL-13), the levels of these cytokines were markedly lower when CXCR4-IL10-MSCs were used. When levels of immunosuppressive cytokines were analyzed, it was observed that TGF-β, PGE_2_ and CCL2 were similarly increased by either MSC type, while IL-10 levels were more markedly increased when CXC4-IL10-MSCs were used (**Figure 2C**). These *in vitro* results reveal that compared to WT-MSCs, CXCR4-IL10-MSCs exhibit increased migration ability towards SDF-1α, as well as enhanced immunosuppressive properties.

### 3.3 CXCR4-IL10-MSCs exert enhanced therapeutic effects against a humanized model of GvHD

**Figure 3:**
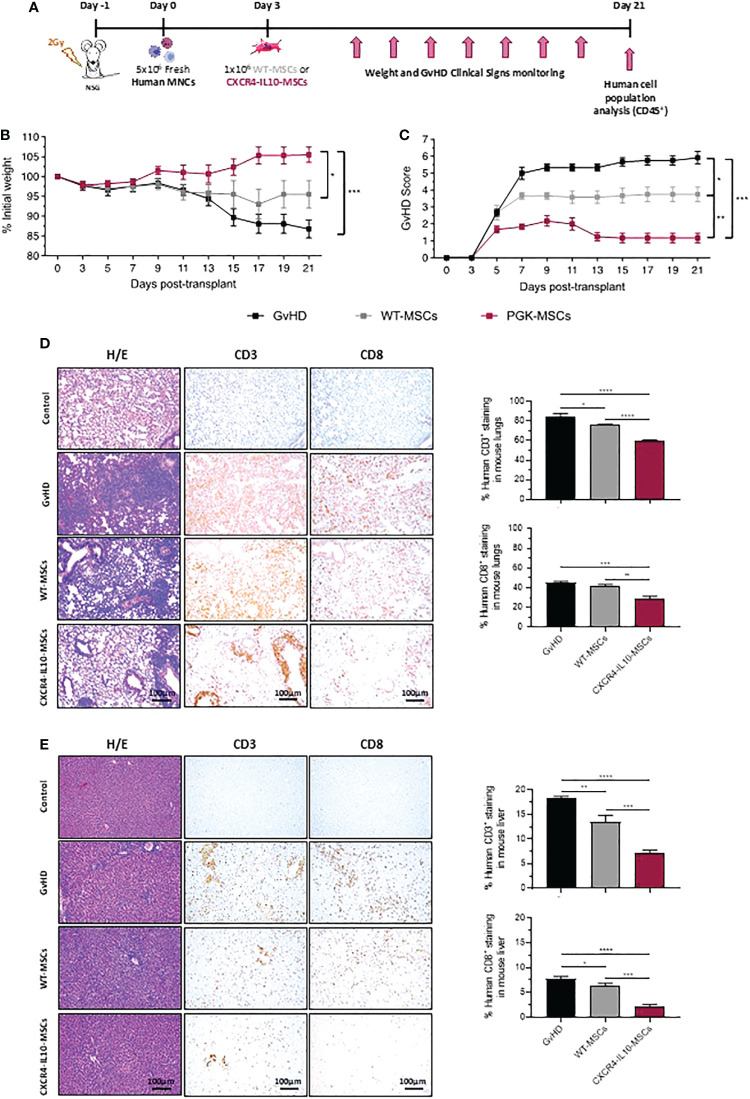
Enhanced therapeutic efficacy of CXCR4-IL10-MSCs in a humanized model of GvHD. **(A)** Schematic diagram of the humanized GvHD model generated in NSG mice. **(B)** Weight kinetics of NSG mice transplanted with hMNCs and treated with WT- or CXCR4-MSCs three days later. **(C)** GvHD clinical score in NSG transplanted recipients treated with WT- or CXCR4-IL10 MSCs. The GvHD score was deduced from analyses of the weight loss, and evaluation of posture, activity, hair texture, skin integrity, and presence of diarrhea of transplanted mice (See materials and methods). **(D)** Histopathological analysis of lung sections from NSG recipients treated with WT- or CXCR4-IL10-MSCs three weeks after NSG mice transplantation. Pictures correspond to representative images of H/E staining and of immunohistochemical staining with anti-hCD3 and anti-hCD8 monoclonal antibodies. Right panels represent the percentage of hCD3^+^ and hCD8^+^ T cells identified in the lung slides. **(E)** Histopathological analysis of liver sections from NSG recipients treated with WT- or CXCR4-IL10-MSCs. Studies are equivalent as those conducted in lungs. Each bar represents the mean ± SEM of data from 5-6 mice per group. *p < 0.05; **p < 0.01; ***p < 0.001; **** p <0.0001. Control: healthy non-treated mice.

To compare the therapeutic effect of WT-MSCs and CXCR4-IL10-MSCs in a humanized GvHD mouse model, immunodeficient NSG mice were irradiated with 2Gy and infused with 5x10^6^ human peripheral blood mononuclear cells (hMNCs) 24h later. Since the weight of transplanted mice started to decrease soon after the infusion of hMNCs as an early sign of GvHD (5% weight decrease in 3 days), recipients were treated with a single dose of either 1x10^6^ WT-MSCs or CXCR4-IL10-MSCs at 3 days post-hMNC transplantation. Additionally, one group of hMNC-transplanted mice remained free from MSCs (GvHD group) as a negative control group (see experimental protocol in **Figure 3A**). Recipients were daily monitored to control the weight and GvHD clinical signs (see GvHD score in **Supplementary Table 1**). As shown in **Figure 3B**, a progressive and marked weight loss was observed in control GvHD mice, which were euthanized 3 weeks after transplantation. The infusion of WT-MSCs limited the weight loss observed in the control GvHD group. Nevertheless, no weight loss was observed in mice treated with CXCR4-IL10-MSCs (**Figure 3B**). Regarding the GvHD clinical score, the control GvHD group showed a high score of 5.76 ± 0.63 (day 21 after hMNC transplantation), which was significantly lower in recipients infused with WT-MSCs (average GvHD score of 3.75 ± 0.95). Remarkably, the GvHD score was further reduced in recipients treated with CXCR4-IL10-MSCs, which never reached a score higher than 2.0, and which was reduced to a very mild GvHD score of 1.17 ± 0.62 on day 21 post-transplantation (**Figure 3C**).

The histopathological GvHD signs were analyzed in different tissues from recipient mice at 21 days post-hMNC transplantation. The H/E staining of skin samples from these animals did not reveal visible alterations in any group of transplanted mice (data not shown). On the contrary, histological analyses of the intestine from the GvHD group showed an almost complete loss of the connective tissue and blood and lymphatic capillaries in the lamina propria of the intestinal villi. In mice treated with either WT-MSCs or CXCR4-IL10-MSCs, a slight decrease in the severity and extension of these lesions was observed, although no major differences were observed between mice receiving either type of MSCs (not shown). H/E staining of lung sections from the GvHD control group revealed abundant infiltrates of human CD3^+^ and CD8^+^ T cells in the parenchyma, accompanied by structure loss due to the formation of large fibrotic zones. In the case of WT-MSC treated mice and more significantly in CXCR4-IL10-MSC treated mice, the severity of these clinical signs was ameliorated as deduced from the significant reduction of hCD3^+^ and hCD8^+^ T cell infiltrates, which lead to a more conserved structure of the parenchyma (see representative pictures and hCD3^+^ and hCD8^+^ analyses in **Figure 3D** **)**. Histopathological analyses of the liver showed a lower damage of this organ compared to the lung. While H/E staining revealed abundant lymphocytic infiltration of the parenchyma, no structure loss or fibrosis was deduced from these studies. After infusion of WT-MSCs and more significantly of CXCR4-IL10-MSCs, the infiltration of the parenchyma by hCD3^+^ and hCD8^+^ cells was significantly reduced, as well as the level of the perivascular inflammation of this organ (see representative images and analyses of human T cells in **Figure 3E**).

Taken together, these results demonstrate that, compared to WT-MSCs, CXCR4-IL10-MSCs are characterized by an enhanced therapeutic efficacy to control the clinical signs of a humanized mouse model of GvHD.

### 3.4 Peripheral blood analyses from mice developing GvHD show that CXCR4-IL10-MSCs enhance the anti-inflammatory response generated by WT-MSCs

**Figure 4:**
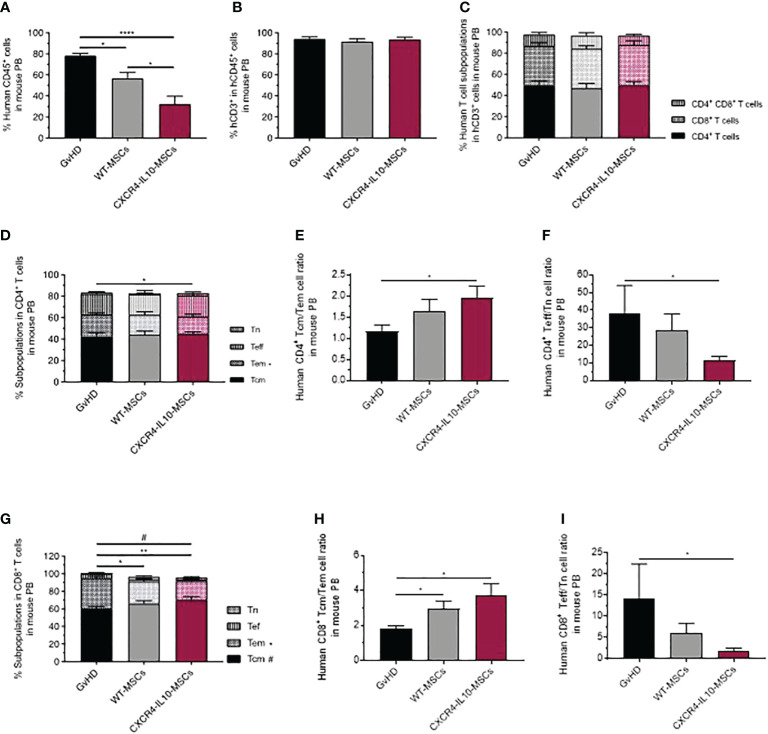
Analysis of peripheral blood human T cells in humanized GvHD recipient mice treated with WT-MSCs or CXCR4-IL10-MSCs. **(A)** Analysis of the proportion of hCD45+ cells present in PB of transplanted NSG recipients and treated with WT- or CXCR4-IL10-MSCs. **(B)** Proportion of hCD3+ cells present in PB hCD45+ cells corresponding to panel **(A)**. **(C)** Composition of PB hCD3+ T cells determined in each group of transplanted mice. **(D)** Frequency of naïve (Tn), effector (Tef), effector memory (Tem) and central memory (Tcm) subpopulations present in PB hCD4+. **(E)** Tcm:Tem ratio and **(F)** Tef:Tn ratio in PB hCD4+ cells. **(G)** Frequency of naïve (Tn), effector (Tef), effector memory (Tem) and central memory (Tcm) subpopulations present in PB hCD8+ T cells. **(H)** Tcm:Tem ratio and **(I)** Tef:Tn ratio in PB hCD8+ cells Analyses were performed 3 weeks after NSG mice transplantation. Each bar represents the mean ± SEM of data from five experiments with NSG mice (n = 15-20 mice per group). *p < 0.05; **p < 0.01; ****p < 0.0001.

At 21 days post-hMNC transplantation, the presence of human leukocytes (hCD45^+^ cells) was first analyzed in mouse peripheral blood (PB). While 78.43 ± 1.97% of PB cells from the GvHD control group consisted of human leukocytes, this value was reduced to 56.58 ± 5.76% in mice infused with WT-MSCs. Moreover, a further decrease to 32.49 ± 7.16% of hCD45^+^ cells was observed in PB of mice treated with CXCR4-IL10-MSCs (**Figure 4A**). In all instances, human leukocytes mainly consisted of CD3^+^ T cells (**Figure 4B** **),** and a similar distribution of hCD4^+^, hCD8^+^ and hCD4^+^CD8^+^ subpopulations was observed in human T cells present in PB of the different study groups (**Figure 4C**). When the distribution of hCD4^+^ T cells was investigated (**Figure 4D**), a similar distribution of naïve T cells (Tn), central memory T cells (Tcm), and effector T cells (Teff) was observed in the three groups. Nevertheless, the proportion of effector memory T cells (Tem) was significantly lower in mice infused with CXCR4-IL10-MSCs. This implied that the ratio of Tcm/Tem hCD4^+^ cells was highest in GvHD mice infused with CXCR4-IL10-MSCs (**Figure 4E**), while the Teff/Tn ratio was significantly lower in this group (**Figure 4F**). When the distribution of hCD8^+^ T cells was investigated, a significant decrease in cells with effector memory phenotype was observed in mice infused with CXCR4-IL10-MSCs (**Figure 4G**). The same trend in Tcm/Tem and Teff/Tn cell ratios observed in hCD4^+^ T cells was also observed in the CD8^+^ T cell population (See **Figures 4H, I**, respectively). These results were confirmed when the absolute numbers of human leukocytes were determined (See **Figures 1SA–E** **)**

The analysis of classical early and late activation markers such as CD25, ICOS, 4-IBB or HLA-DR in PB hCD3^+^ T cells showed a significant increase of CD25^+^ cells in mice infused with CXCR4-IL10-MSCs, but not with WT-MSCs, compared with the control GvHD group (**Figure S2A** **)**. This difference was accounted by increases of CD4^+^CD25^+^ cells (**Figure S2B**), while no differences were noted in any of the CD8^+^ cell subpopulations (**Figure S2C**). When inhibition and exhaustion markers (CTLA4, PD1, TIGIT or TIM3) were analyzed in hCD3^+^ T cells (**Figure S2D**) and in hCD4^+^ or hCD8^+^ T cell subpopulations (**Figure S2E, F**, respectively), only a significant increase in the proportion of CD4^+^ TIGIT^+^ cells was observed in GvHD mice treated with CXCR4-IL10-MSCs compared to the GvHD group (**Figure S2E**).

**Figure 5:**
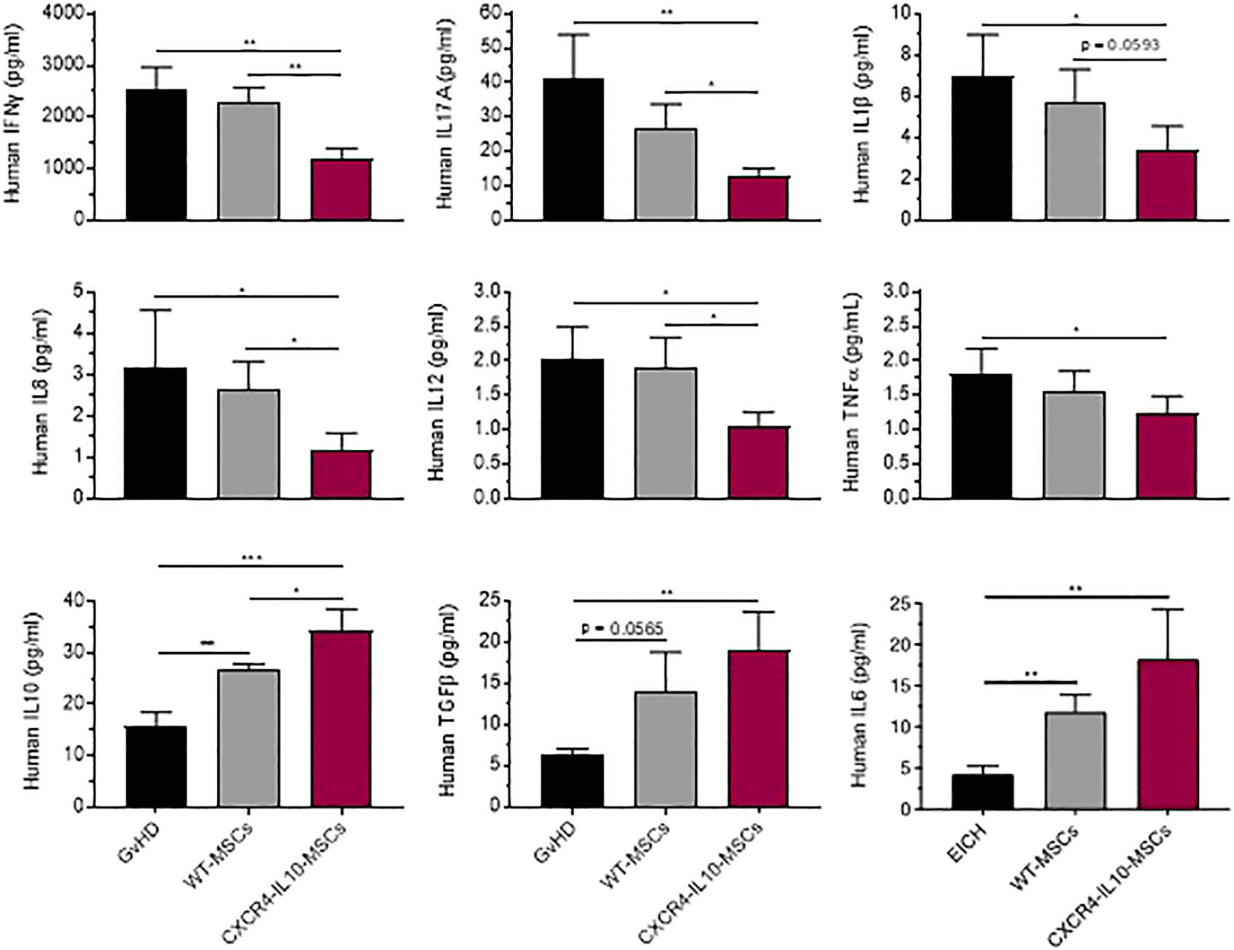
Enhanced changes of immunomodulatory profile in the serum of GvHD recipients treated with CXCR4-IL10-MSCs. Analysis of cytokine and factor levels in the serum of NSG recipients treated with WT- or CXCR4-IL10-MSCs. Analyses were conducted three weeks after NSG mice transplantation. Each bar represents the mean ± SEM of data from 9-12 mice per group. *p < 0.05; **p < 0. 01; ***p < 0.001.

In subsequent experiments, levels of human cytokines involved in GvHD were analyzed in the serum of the three groups of transplanted mice. As shown in **Figure 5**, levels of pro-inflammatory human cytokines (IFNγ, IL-17A, IL-1β, IL-8, IL-12 and TNFα) were lower in WT-MSC treated mice compared with the control GvHD group. Nevertheless, these levels were significantly lower in mice treated with CXCR4-IL10-MSCs compared to WT MSC-treated mice. When levels of soluble factors involve in the immunosuppression properties of MSCs, such as IL-10, TFGb and IL-6, were analyzed, a significant increase was observed in WT-MSC treated mice with respect the GvHD control, although this difference was more marked in CXCR4-IL10-MSCs treated mice (31–33).

### 3.5 The analysis of spleen from mice with GvHD shows that CXCR4-IL10-MSCs induce an enhanced anti-inflammatory response compared to WT WSCs

**Figure 6:**
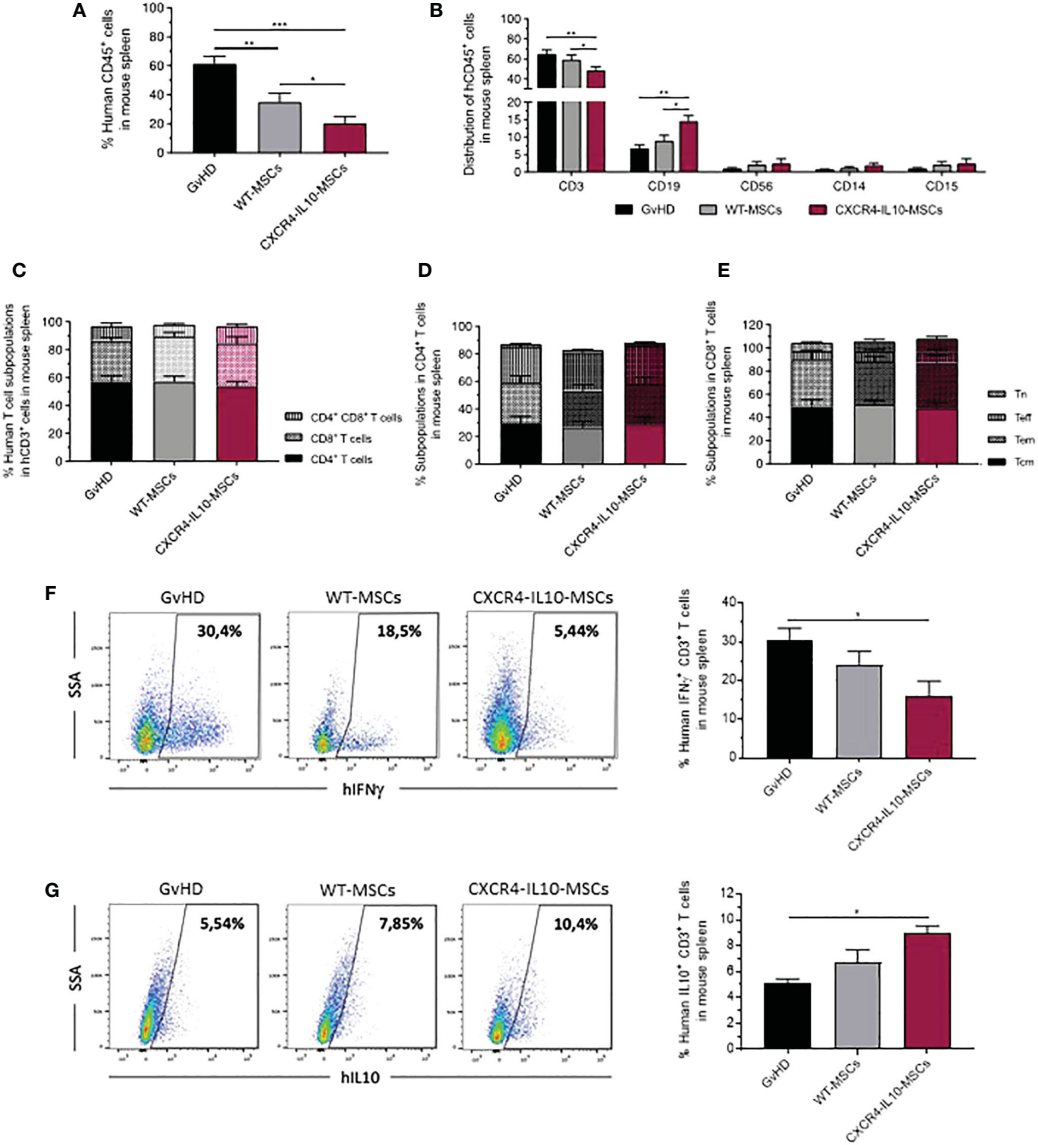
Analysis of human T cells in the spleen of humanized GvHD recipients treated with WT-MSCs or CXCR4-IL10-MSCs. **(A)** Percentage of hCD45^+^ cells infiltrating in the spleen of transplanted NSG recipients treated with WT- or CXCR4-IL10-MSCs. **(B)** Composition of hCD45^+^ cells characterized in panel **(A)**. **(C)** Distribution of CD4^+^, CD8^+^ and CD4^+^CD8^+^ cells present in the T cells determined in panel **(B)**. **(D)** Frequency of naïve (Tn), effector (Teff), effector memory (Tem) and central memory (Tcm) subpopulations present in hCD4^+^ and **(E)** hCD8^+^ T cells. **(F)** Analysis of the percentage of Th1 cells (CD3^+^ IFNγ^+^ T cells) and **(G)** Th2 T cells (CD3^+^ IL10^+^ cells) present in the spleen of transplanted recipients. Analyses were conducted three weeks after transplantation of NSG recipients. Each bar represents the mean ± SEM corresponding to 10-20 mice per group. *p < 0.05; **p < 0.01; ***p < 0.001.

As observed in peripheral blood, the infusion of WT-MSCs induced a significant decrease in the proportion of human leukocytes present in the spleen of hMNC-transplanted mice, compared with the GvHD control group. Once again, a more marked decrease of human leukocytes was observed in the spleen of recipients treated with CXCR4-IL10-MSCs (**Figure 6A**). When the distribution of hCD45^+^ cells was analyzed, a significant decrease of hCD3^+^ T cells was observed in CXCR4-IL10-MSCs treated mice, compared with the control GvHD group, and also with WT-MSC treated mice. Concomitantly, the proportion of hCD19^+^ B cells present in hCD45^+^ cells was higher in CXCR4-IL10-MSCs treated mice, compared with the other two groups (**Figure 6B** **)**. Similar conclusions were obtained when the absolute numbers of CD19^+^ cells were determined (**Figure S1F**). No significant differences in the percentage of NK cells (CD56^+^), monocytes (CD14^+^) and granulocytes (CD15^+^) (**Figure 6B** **)**, nor in the distribution of splenic human T cell subpopulations among the three groups of transplanted mice were observed (**Figures 6C–E**). When the early and late activation markers were analyzed in human T cells present in the spleen of these mice, WT-MSCs and more markedly CXCR4-IL10-MSCs increased the proportion of CD4^+^CD25^+^T cells, compared with the control GvHD group (**Figure S3A**, **2B**). Analyses of the exhaustion markers only showed a significant increase in infiltrating TIM3^+^ cells both in total CD3^+^ T cells and also in CD4^+^ and CD8^+^ T cells subpopulations from mice treated with CXCR4-IL10-MSCs, compared to the control GvHD group **(** **Figures S3D–F** **)**.

Studies of the polarization of human T cells present in the spleen of transplanted NSG mice were conducted by means of the analysis of the intracellular expression of the pro-inflammatory IFNγ and the anti-inflammatory IL-10 cytokines, as described in Materials and Methods. As shown in **Figure 6F**, the infusion of WT-MSCs slightly decreased the percentage of IFNγ-secreting T cells compared with the control GvHD group. Nevertheless, this decrease became statistically significant in mice treated with CXCR4-IL10-MSCs. On the contrary, a moderate, non-significant increase in the percentage of IL10-secreting T cells was observed in WT-MSC treated mice, which became statistically significant in the CXCR4-IL10-MSC group (**Figure 6G** **)**.

To obtain a more comprehensive view of the immune pathways involved in the GvHD response, RNA-seq analyses were performed in hCD45^+^ cells isolated from the spleen of transplanted mice. Comparative Gene Set Enrichment Analysis (GSEA) between the control GvHD group and the WT-MSC group **(** **Figure S4A** **)** revealed an enrichment of genes overexpressed in cells with anti-inflammatory phenotype (Th2 and Treg cells) as compared with cells with a pro-inflammatory phenotype (Th1, Th17 or effector T cells) after MSC infusion. On the other hand, even though CXCR4-IL10-MSC group only showed marked anti-inflammatory phenotype when Treg and effector T cells were compared **(** **Figure S4B** **)**, when WT-MSCs were compared with the ones treated with CXCR4-IL10-MSCs, it was found that human T cells from mice that received CXCR4-IL10-MSCs presented an enrichment in the expression of an anti-inflammatory profile **(** **Figure S4C**). Because global gene expression analyses suggested that CXCR4-IL10-MSCs significantly enhanced anti-inflammatory polarization of T cells infiltrating the spleen, this polarization was investigated in more detail in a panel of inflammatory and anti-inflammatory cytokines by qRT-PCR analyses (see Material and Methods). These analyses confirmed a significant reduction in the relative expression of pro-inflammatory factors (*IFNγ*, *IL17* and *IL22*; **Figure S4D** top), as well as an increase in the expression of immunoregulatory factors (*FoxP3*, *IL5* and *IL10*; **Figure S4D** bottom). Interestingly, increases of *FoxP3* expression associated with CXCR4-IL10-MSCs were consistent with increases in the percentage of CD45^+^CD3^+^CD4^+^CD25^+^ T cells shown in **Figure S3B**.

These data indicated that the spleen from GvHD recipients treated with CXCR4-IL10-MSCs presented enhanced anti-inflammatory polarization of human T cells compared with the effects induced by WT-MSCs.

### 3.6 CXCR4-IL10-MSCs, but not WT-MSCs, inhibited the human B cell differentiation and increased the proportion of IL10-secreting regulatory B cells in the spleen of humanized GvHD recipients

**Figure 7:**
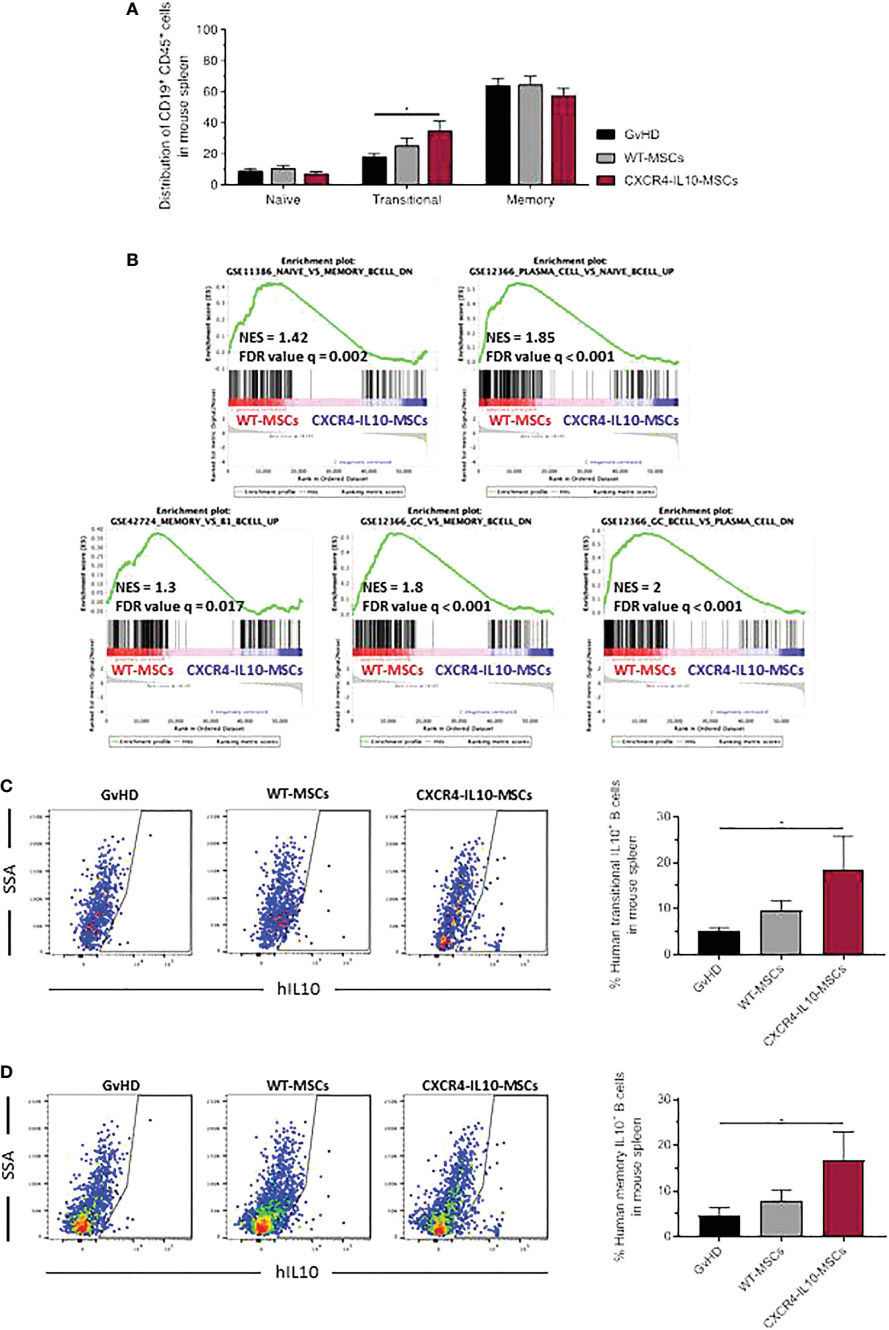
Characterization of human CD19^+^ B-cell subpopulations in the spleen of humanized GvHD recipients treated with WT-MSCs or CXCR4-IL10-MSCs. **(A)** Flow cytometry phenotype of spleen-infiltrating human CD19^+^ B-cell subpopulations in GvHD recipients treated with WT-MSCs or CXCR4-IL10-MSCs. **(B)** RNA sequencing of human CD45^+^ cells isolated from the spleen of WT-MSC-treated mice relative to the CXCR4-IL10-MSC-treated group by gene cluster enrichment (GSEA) of immune signatures corresponding to B cells. **(C)** Representative flow cytometry analysis of each transplanted group and representation of the percentage of IL10^+^ transitional B cells, analyzed as CD45^+^CD19^+^CD24^low/+^ and CD38^+^CD27^+-^IL10^+^ cells. **(D)** Similar analyses as those conducted in panel C, showing the percentage of IL10^+^ memory B cells, analyzed as CD45^+^CD19^+^CD27^+^IL10^+^ cells. Each analysis compares data corresponding to 3 mice per group. NES, Normalized Enrichment Score; FDR, False Discovery Rate. *p < 0.05.

In these studies, we first observed that neither the population of naïve B cells (CD45^+^CD19^+^CD24^-^CD38^-^CD27^-^), nor memory B or plasma cells (CD45^+^CD19^+^CD27^+^) showed differences among the three groups of transplanted mice **(** **Figures 7A** and **S1G** **)**. With respect to the transitional B cell population (CD45^+^CD19^+^CD24^low/+^CD38^+^CD27^-^) present in the spleen of these animals, a modest increase was observed in mice treated with WT-MSCs, while a significant increase was noted in CXCR4-IL10-MSC treated mice **(** **Figures 7A** and **S1G** **)**. To investigate at the gene expression level if WT or CXCR4-IL10-MSCs affected the differentiation of B cells present in the spleen of transplanted mice, GSEA were conducted. When compared with the control GvHD group, recipients treated with any type of MSCs presented an enrichment of genes expressed in naïve or transitional B cells, with respect to terminally differentiated B cells (**Figures S5A, B**). More remarkably, when both types of Ad-MSCs-treated groups were compared, hCD45+ cells isolated from the spleen from WT-MSC group showed enrichment in genes overexpressed in memory or plasmatic B cells, while CXCR4-IL10-MSC-treated mice displayed enrichment in transcriptional profiles of immature B cells, including naïve or transitional (B1 or germinal center) B cells (**Figure 7B**). Analyses of the intracellular staining of IL-10 in transitional and plasma B cell subpopulations revealed that the percentage of IL-10^+^ Bregs present in these populations was statistically higher in recipients treated with CXCR4-IL10-MSCs **(** **Figures 7C, D** **).**

These results suggested that CXCR4-IL10-MSCs enhance the maintenance B cell populations in a transition state which did not complete their differentiation to memory B cells or plasma cells. Moreover, our data reveal that compared to WT-MSCs, CXCR4-IL10-MSCs not only favor the development of T cells with an immunoregulatory phenotype, but also of IL10-secreting B cells that should contribute to GvHD modulation.

## 4 Discussion

In the current study, a bicistronic LV carrying codon-optimized versions of *CXCR4* and *IL10* cDNAs was generated to facilitate the stable expression of these two molecules in hMSCs. Our first *in vitro* analyses showed that CXCR4-IL10-MSCs retain the characteristics of WT-MSCs (30) while exhibit enhanced ability to migrate towards SDF-1α, an observation that is consistent with previous studies indicating that the ectopic expression of CXCR4 enhances the migration of MSCs to inflammed tissues (27). Similarly, our *in vitro* data revealing that CXCR4-IL10-MSC inhibits the proliferation of T cells is sustained by the observation that compared to WT-MSCs, CXCR4-IL10-MSC co-cultures show marked decreases in levels of IFNγ and TNFα, together with increased levels of IL-10 (34).

Since GvHD remains one of the leading causes of mortality associated with alloHSCT, we investigated whether CXCR4-IL10-MSCs improved the moderate anti-GvHD therapeutic potential of WT-MSCs. Studies conducted in a clinically relevant GvHD model evidenced a marked reduction of the GvHD score in recipients treated with CXCR4-IL10-MSCs, as compared with the WT-MSC group. Significantly, the clinical status of recipients infused with either WT- or CXCR4-IL10-MSCs was consistent with analyses of T cell infiltration in the GvHD target tissues. Remarkably, numbers of hCD3^+^ and hCD8^+^ T cells in lungs and liver from GvHD mice treated with CXCR4-IL10-MSCs were significantly lower compared with numbers observed in the WT-MSC group. This observation was associated with the improved tissue architecture of these organs, and also with the reduced proportion of hCD4^+^ and hCD8^+^ T cells in PB. Additionally, our data showed that CXCR4-IL10-MSCs significantly increased the balance of anti-inflammatory *versus* inflammatory cytokines in PB, and increased the proportion of circulating CD45^+^CD3^+^CD4^+^CD25^+^ immunoregulatory T cells. All these results are consistent with previous studies in which the effects of MSCs on GvHD were investigated (35), and confirm the improved therapeutic efficacy of CXCR4-IL10-MSCs to modulate the disease.

Studies conducted in the spleen of transplanted mice also showed a lower expansion of human T cells in CXCR4-IL10-MSC-treated mice compared with the WT-MSC group. Additionally, analyses of human T cell polarization in this organ showed that CXCR4-IL10-MSCs markedly reduced the presence of pro-inflammatory IFNγ^+^ human T cells, while increased numbers of anti-inflammatory IL10^+^ T cells compared with the WT-MSC group. Moreover, the increased expression of the transcription factor *FoxP3* in the spleen of CXCR4-IL10-MSC-treated mice revealed the enhanced effect of these cells to induce T cell polarization towards Th2 and Treg cells. These studies also showed that compared to the effects mediated by WT-MSCs, CXCR4-IL10-MSCs increased numbers of splenic transitional B cells, suggesting a blockage in the differentiation of B cells to memory B or plasma cells. Additionally, analyses of IL10-secreting transitional or memory B cells showed that these regulatory B cell populations were increased after the infusion of WT-MSCs, although once again, a significantly higher increase was associated to the infusion of CXCR4-IL10-MSCs. These results are thus consistent with studies from Peng et al., who described that Bregs were decreased in patients with active GvHD (13), and suggest that increased levels of these immunoregulatory populations might play a role in the anti-GvHD effects of MSCs, and more significantly of CXCR4-IL10-MSCs.

Recent clinical studies have shown that MSCs can mediate not only short-term, but also long-term responses in patients with steroid-refractory GvHD (36). Although we ca hypothesize that single or multiple infusions of CXCR4-IL10-MSCs might also exert improved long-term responses in patients with either acute or chronic GvHD, further experimental studies will be required to confirm this hypothesis. Additionally, although no side effects have been observed in our study, additional biodistribution and safety studies are currently being conducted with the aim of confirming that, as shown with WT MSCS, CXCR4-IL10-MSCs constitute a safe medicinal product with enhanced therapeutic properties.

Taken together, our study demonstrates that compared with WT-MSCs, CXCR4-IL10-MSCs exert a superior therapeutic activity in a humanized GvHD mouse model. Our data also reveals that this improved therapeutic benefit is associated with a marked inhibition of a pro-inflammatory environment in recipients that favors the proliferation of regulatory immune populations. Overall, these results strongly suggest that the stable ectopic expression of CXCR4 and IL-10 characteristic of CXCR4-IL10-MSCs should improve the therapeutic efficacy previously characterized in WT-MSCs in alloHSC-transplanted patients developing GvHD.

## Data availability statement

The datasets discussed in this publication have been deposited in NCBI's Gene Expression Omnibus and are accessible through GEO Series accession number GSE223390 (https://www.ncbi.nlm.nih.gov/geo/query/acc.cgi?acc=GSE223390).

## Ethics statement

The animal study was reviewed and approved by CIEMAT Animal Experimentation Ethical Committee according to the approved biosafety and bioethics guidelines (Protocol number: PROEX 252-19).

## Author contributions

RH-S, conducted experiments and prepared a manuscript draft; MF-G, conducted experiments and revised the manuscript; MH-R, conducted experimental work; CS-C, performed histological and RNA expression analysis; JB and RY designed the study and prepared final manuscript. All authors contributed to the article and approved the submitted version.

## Supplementary material

Supplementary Figure 1 Absolute numbers of the different human cell types present in peripheral blood and spleen of GvHD recipients treated with WT-MSCs or CXCR4-IL10-MSCs. Samples from peripheral blood were analyzed by flow cytometry to calculate the absolute number of hCD45^+^ cells **(A)**, hCD3^+^ cells **(B)**, T cell subpopulation **(C)** and subset of hCD4^+^ cells **(D)** and hCD8^+^ cells **(E)**. Samples from spleen were analyzed by flow cytometry to calculate the absolute number of hCD19^+^ cells **(F)** and subtypes of hCD19^+^ cells **(G)**. Data were analyzed 3 weeks after transplantation of hMNCs and treated with WT-MSCs or CXCR4-IL10-MSCs. Each bar represents the mean ± SEM. *p < 0.05; **p < 0.01.

Supplementary Figure 2 Analysis of the activation and exhaustion markers of human T cells present in peripheral blood of GvHD recipients treated with WT-MSCs or CXCR4-IL10-MSCs. **(A)** Activation profile of human T cells, analyzed as CD45^+^CD3^+^ cells, in PB. **(B)** Activation profile of hCD4^+^ T cells. **(C)** Activation profile of hCD8^+^ T cells. Exhaustion profile of CD3^+^ **(D)**, CD4^+^ **(E)** and CD8^+^ **(F)** human T cells present in PB of NSG recipients. Data were analyzed 3 weeks after transplantation of hMNCs and treated with WT-MSCs or CXCR4-IL10-MSCs. Each bar represents the mean ± SEM (n=10-12 mice per group). *p < 0.05; **p < 0.01.

Supplementary Figure 3 Analysis of the activation and exhaustion markers of human T cells present in the spleen of GvHD recipients treated with WT-MSCs or CXCR4-IL10-MSCs. **(A)** Activation profile of CD3^+^T cells present in the spleen of transplanted recipients. **(B)** Activation profile of hCD4^+^ T cells. **(C)** Activation profile of hCD8^+^ T cells. Exhaustion profile of CD3^+^ **(D)**, CD4^+^ **(E)** and CD8^+^ **(F)** human T cells present in spleen of NSG recipients. Data were analyzed 3 weeks after transplantation of hMNCs and treated with WT-MSCs or CXCR4-IL10-MSCs. Each bar represents the mean ± SEM (n=10-12 mice per group). *p < 0.05.

Supplementary Figure 4 Transcriptome analysis of human CD45^+^ cells isolated from the spleen of GvHD recipients treated with WT- or CXCR4-IL10-MSCs by gene cluster enrichment (GSEA) of T cell immune signatures. Plots show enrichment analyses in T cell immune signatures in the GvHD group compared to the WT-MSC **(A)** or the CXCR4-IL10-MSCs **(B)** group. **(C)** Gene enrichment analysis comparing the WT- with the CXCR4-IL10-MSCs group. **(D)** qRT-PCR mRNA analyses of cytokines involved in the polarization of human T cells. Data were analyzed 3 weeks after transplantation (n = 3 mice/group). NES = Normalized Enrichment Score; FDR = False Discovery Rate. *p < 0.05; ***p < 0.001.

Supplementary Figure 5 Transcriptome analysis of human CD45^+^ cells isolated from the spleen of GvHD recipients treated with WT- or CXCR4-IL10-MSCs by gene cluster enrichment (GSEA) of B cells immune signatures. Plots show enrichment analysis in B cell immune signatures in the GvHD control group compared to **(A)** WT-MSCs group and **(B)** CXCR4-IL10-MSCs group. n = 3 mice/group. Data were analyzed 3 weeks after transplantation. NES = Normalized Enrichment Score; FDR = False Discovery Rate.
