# Supplementary material for: Improved efficacy of mesenchymal stromal cells stably expressing CXCR4 and IL-10 in a xenogeneic graft versus host disease mouse model
Source: Front Immunol. 2023 Feb 1;14:1062086. doi: 10.3389/fimmu.2023.1062086 (PMC9929539; doi:10.3389/fimmu.2023.1062086)
Supplement: Supplementary file 1 [file DataSheet_1.pdf]

## Supplementary Table 1

| Target | Direction | Sequence 5'-3' |
|---|---|---|
| Psi | Forward | CAGGACTCGGCTTGCTGAAG |
| Psi | Reverse | TCCCCCGCTTAATACTGACG |
| Psi | Probe | [FAM]-CGCACGGCAAGAGGCGAGG |
| Alb | Forward | GCTGTCATCTCTTGTGGGCTG |
| Alb | Reverse | ACTCATGGGAGCTGCTGGTTC |
| Alb | Probe | [VIC]-CCTGTCATGCCCACACAAATCTCTCC |

**Supplementary Table 1.** Sequences of primers and probes used to determine the Vector Copy Number. Psi = packaging sequence of the provirus, Alb = human albumin gene, used as normalizer.

**Supplementary Table 2**

| Grade | Weight Loss | Diarrhea | Posture | Activity | Fur Texture | Skin Integrity |
|---|---|---|---|---|---|---|
| 0 | < 10% | No | Normal | Normal | Normal | Normal |
| 1 | > 10% to < 25% | Yes | Hunching at rest | Mild to moderate decrease | Mild to moderate rufflings | Scaling of paw and tails |
| 2 | > 25% | Yes | Severely hunching | Severe decrease | Severe rufflings | Obviously denuded |

**Supplementary Table 2.** GvHD clinical signs scoring parameters. GvHD score was established using a grading scale of 0 (non-GvHD) to 2 (severe GVHD) for each parameter listed in the table. Total GvHD grade of each mouse was the sum of degrees obtained in each parameter.

## Supplementary Table 3

| Human Hematopoietic Linage Characterization | | | | | |
|---|---|---|---|---|---|
| Antigen | Reactivity | Fluorophore | Vendor | Catalog | Clone |
| CD45 | Human | APC | BioLegend | 368518 | HI30 |
| CD3 | Human | PE-Cy5 | BioLegend | 300410 | UCHT1 |
| CD19 | Human | PE | Beckman Coulter | IM1285U | J4.119 |
| CD14 | Human | BV711 | BioLegend | 301838 | M5E2 |
| CD15 | Human | PE-Cy7 | BD Bioscience | 560827 | HI98 |
| CD56 | Human | APC-Cy7 | BioLegend | 318332 | HDC56 |

| Human T cell Characterization | | | | | |
|---|---|---|---|---|---|
| Antigen | Reactivity | Fluorophore | Vendor | Catalog | Clone |
| CD45 | Human | BV711 | BioLegend | 304050 | HI31 |
| CD3 | Human | PE-Cy7 | BioLegend | 300420 | UCHT1 |
| CD4 | Human | PE-Cy5 | BioLegend | 317412 | OKT4 |
| CD8 | Human | PE | BioLegend | 344706 | B9.11 |
| CD45RA | Human | APC-FIRE | BioLegend | 304151 | HI100 |
| CD27 | Human | PerCP-Cy5.5 | BioLegend | 393210 | QA17A18 |
| CD62L | Human | FITC | Beckman Coulter | IM1231 | Dreg 56 |

**Supplementary Table 3.** Monoclonal antibodies used for the characterization of the human hematopoietic lineage and human T cell population in peripheral blood and spleen of NSG mice.

**Supplementary Table 4**

| Human T cell activation markers | | | | | |
|---|---|---|---|---|---|
| Antigen | Reactivity | Fluorophore | Vendor | Catalog | Clone |
| CD45 | Human | BV711 | BioLegend | 304050 | HI31 |
| CD3 | Human | APC-Cy7 | BioLegend | 100222 | 17A2 |
| CD4 | Human | APC | Miltenyi Biotec | 130-091-232 | MT466 |
| CD8 | Human | PE | BioLegend | 344706 | B9.11 |
| CD69 | Human | PE-Cy7 | BD Bioscience | 335792 | L78 |
| CD25 | Human | PE-Cy5 | BD Bioscience | 555433 | M-A252 |
| HLA-DR | Human | FITC | BD Bioscience | 555558 | TÜ39 |
| CD45 | Human | BV711 | BioLegend | 304050 | HI31 |
| CD3 | Human | FITC | Inmunotech | A07746 | UCHT1 |
| CD4 | Human | PE-Cy5 | BioLegend | 317412 | OKT4 |
| CD8 | Human | PE | BioLegend | 344706 | B9.11 |
| 4-IBB (CD137) | Human | APC | BioLegend | 309809 | 4B4-1 |
| ICOS (CD278) | Human | PerCP-Cy5.5 | BioLegend | 313518 | C398.4A |

| Human T cell exhaustion markers | | | | | |
|---|---|---|---|---|---|
| Antigen | Reactivity | Fluorophore | Vendor | Catalog | Clone |
| CD45 | Human | BV711 | BioLegend | 304050 | HI31 |
| CD3 | Human | FITC | Inmunotech | A07746 | UCHT1 |
| CD4 | Human | PE-Cy5 | BioLegend | 317412 | OKT4 |
| CD8 | Human | PE | BioLegend | 344706 | B9.11 |
| CTLA4 (CD152) | Human | APC | BioLegend | 349908 | L3D10 |
| PD1 (CD279) | Human | PerCP-Cy5.5 | BioLegend | 329913 | EH12.2H 7 |
| TIGIT | Human | PE-Cy7 | BioLegend | 372713 | A15153G |
| TIM3 (CD366) | Human | APC-Cy7 | BioLegend | 345025 | F38-2E2 |

**Supplementary Table 4.** Monoclonal antibodies used for the analysis of human T cell activation and exhaustion markers in peripheral blood and spleen of NSG mice.

**Supplementary Table 5**

| Human T cell polarization | | | | | |
|---|---|---|---|---|---|
| Antigen | Reactivity | Fluorophore | Vendor | Catalog | Clone |
| CD45 | Humano | APC-Cy7 | BioLegend | 304014 | HI30 |
| CD3 | Humano | FITC | Inmunotech | A07746 | UCHT1 |
| IFNy | Humano | PE-Cy7 | BD Bioscience | 557643 | 4SB3 |
| CD45 | Humano | APC-Cy7 | BioLegend | 304014 | HI30 |
| CD3 | Humano | FITC | Inmunotech | A07746 | UCHT1 |
| IL10 | Humano | PE-Cy7 | BioLegend | 557643 | JES3-9D7 |

| Human B cell characterization | | | | | |
|---|---|---|---|---|---|
| Antigen | Reactivity | Fluorophore | Vendor | Catalog | Clone |
| CD45 | Humano | BV711 | BioLegend | 304050 | HI31 |
| CD19 | Humano | PE-Cy5 | BioLegend | 302210 | HIB19 |
| CD27 | Humano | PerCP-Cy5.5 | BioLegend | 393210 | QA17A18 |
| CD24 | Humano | PE | BD Bioscience | 302210 | ML5 |
| CD38 | Humano | FITC | BD Bioscience | 555459 | HIT2 |
| IL10 | Humano | PE-Cy7 | BioLegend | 557643 | JES3-9D7 |

**Supplementary Table 5.** Surface and intracellular monoclonal antibodies used for the analysis of human T cell polarization and human B cell characterization in the spleen of NSG mice.

**Supplementary Table 6**

| Primary Antibodies | | | | | |
|---|---|---|---|---|---|
| Antigen | Reactivity | Vendor | Catalog | Clone | Dilution |
| CD3 | Human in rabbit | Dako Agilent | GA503 | Policlonal | - |
| CD8 | Human in mouse | Dako Agilent | M7103 | C8/144B | 1:50 |
| Secondary Antibodies | | | | | |
| Rabbit IgG | Rabbit in donkey | Jackson | 715-065-151 | - | 1:1000 |
| Mouse IgG | Mouse in donkey | Jackson | 711-065-152 | - | 1:1000 |

**Supplementary Table 6.** Primary and secondary antibodies used in immunohistochemistry performed on skin, small intestine, lungs and liver, typical GvHD target organs.
