# Supplementary figures and images for: Improved efficacy of mesenchymal stromal cells stably expressing CXCR4 and IL-10 in a xenogeneic graft versus host disease mouse model
Source: Front Immunol. 2023 Feb 1;14:1062086. doi: 10.3389/fimmu.2023.1062086 (PMC9929539; doi:10.3389/fimmu.2023.1062086)

Figure S1

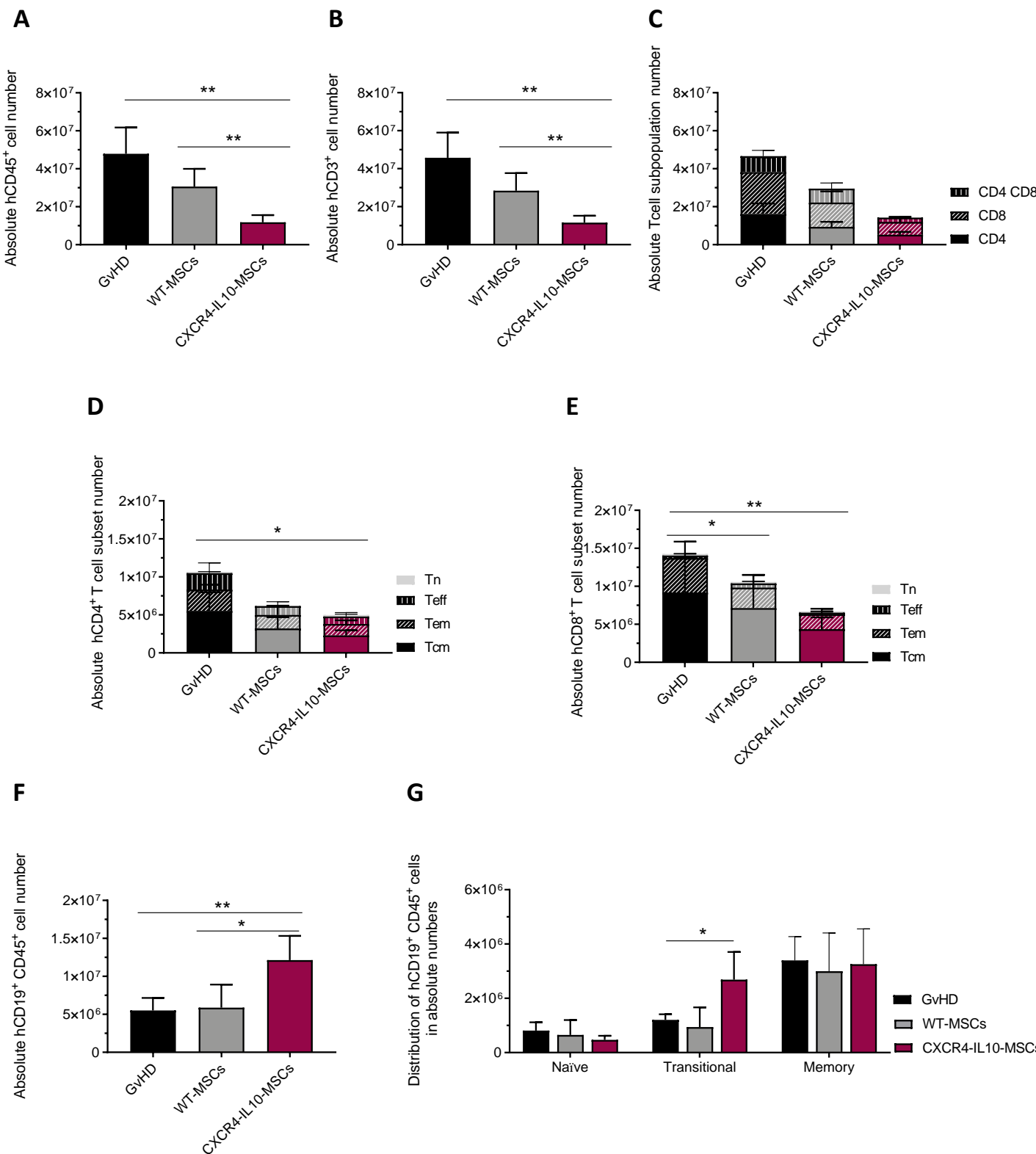

Figure S2

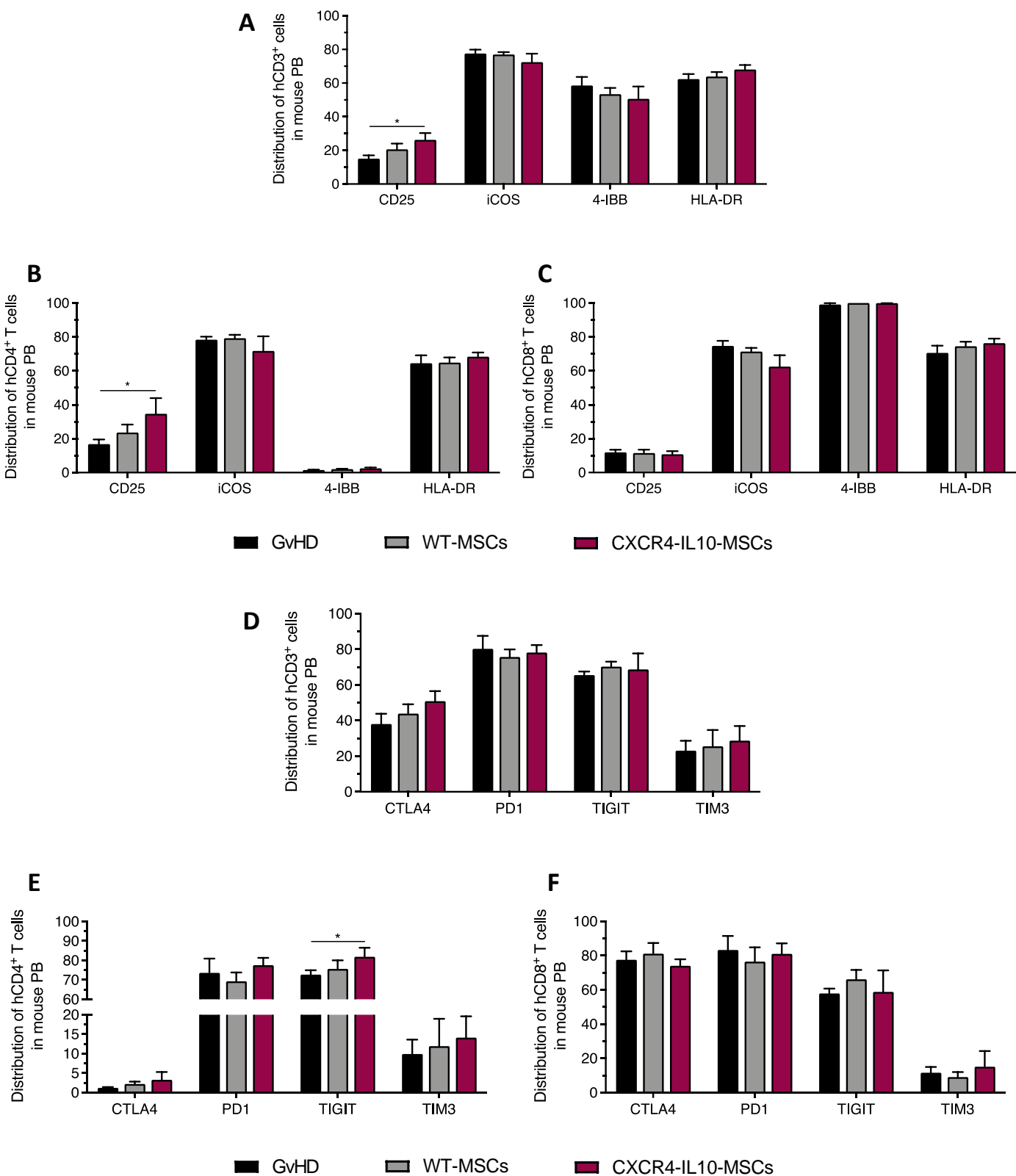

Figure S3

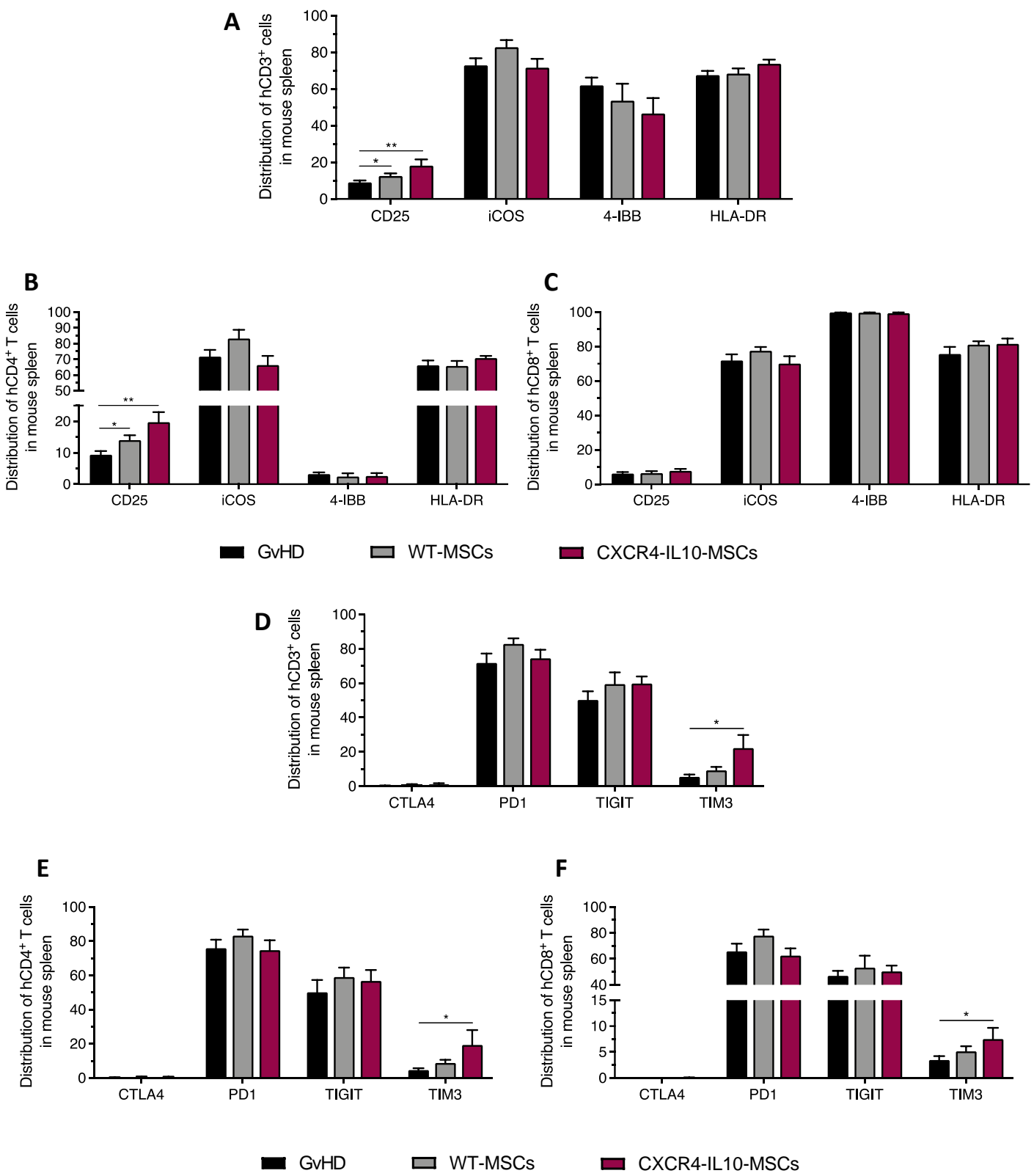

Figure S4

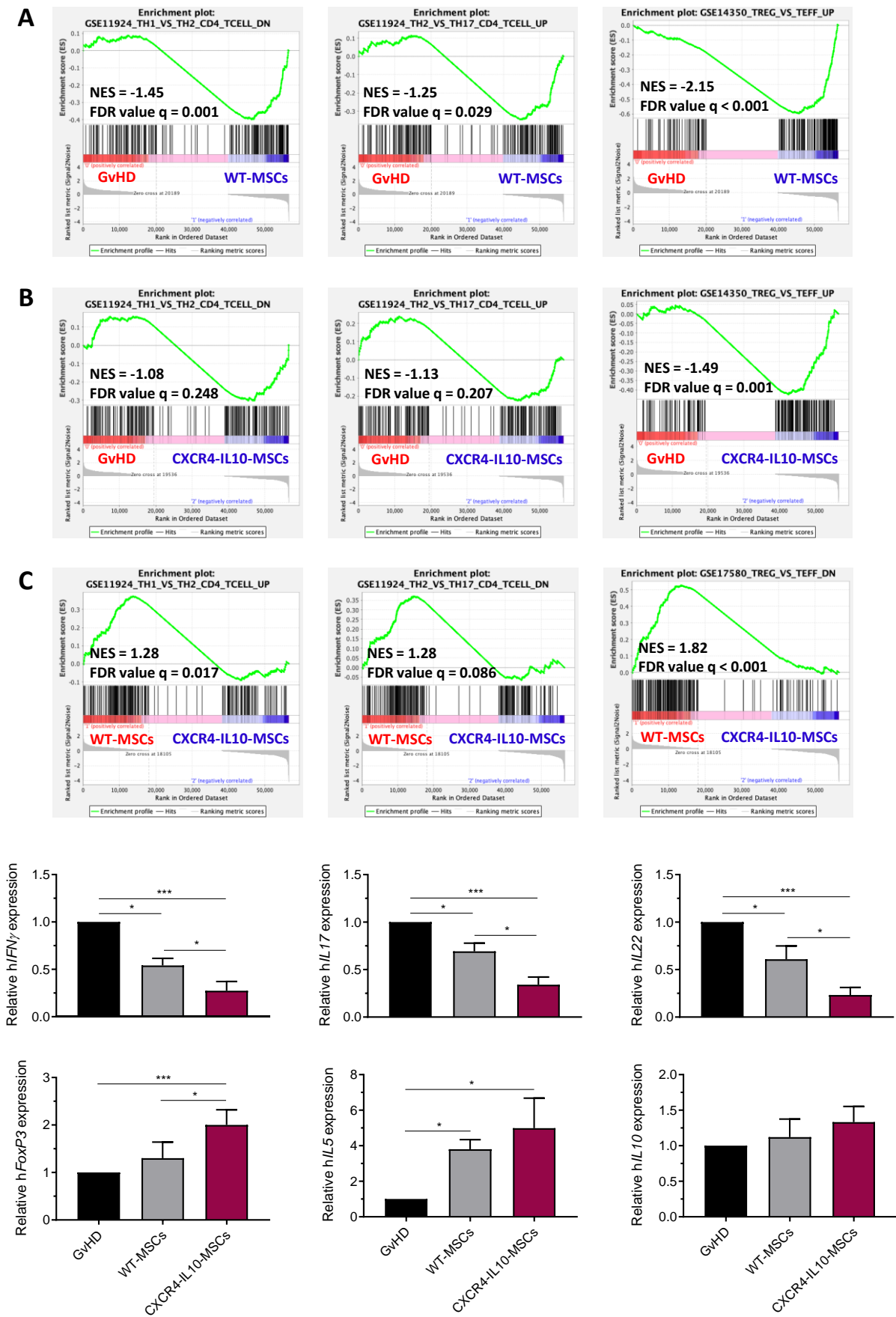

Figure S5

A

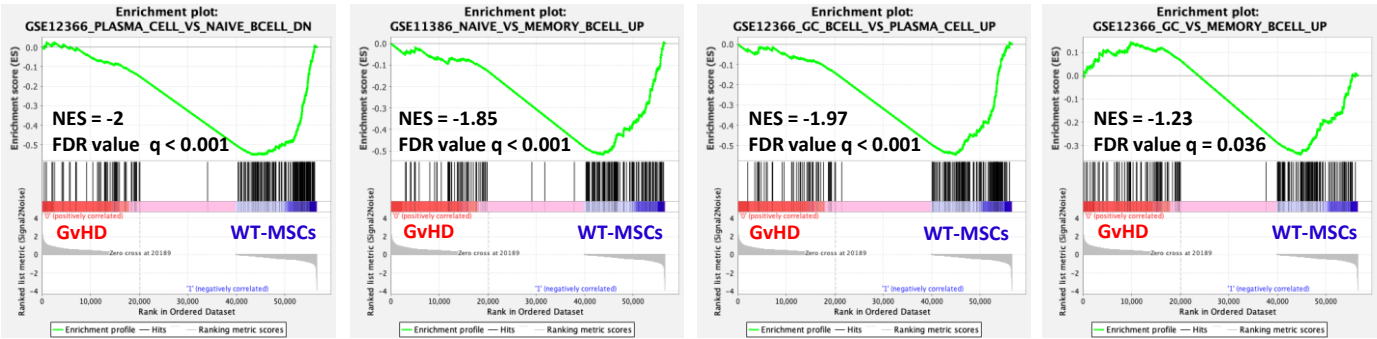

B

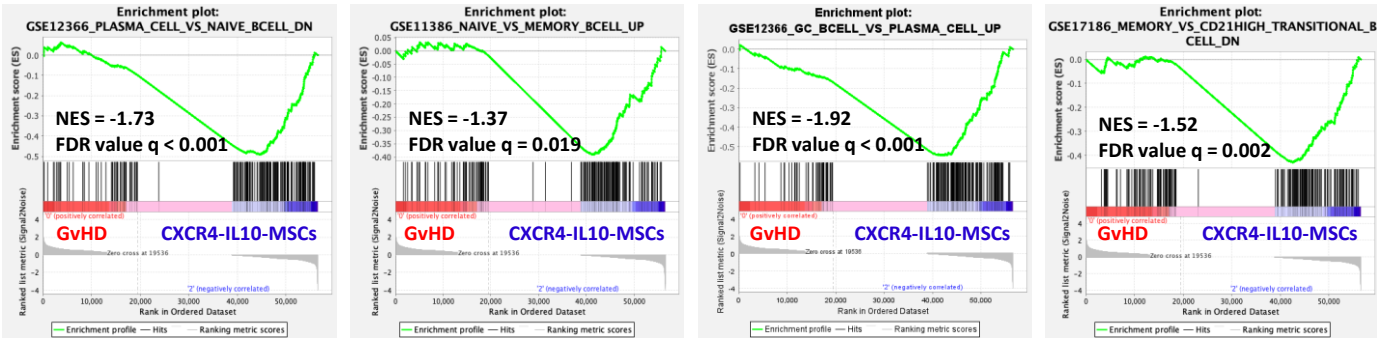

Supplement: Supplementary Figure 1 — Absolute numbers of the different human cell types present in peripheral blood and spleen of GvHD recipients treated with WT-MSCs or CXCR4-IL10-MSCs. Samples from peripheral blood were analyzed by flow cytometry to calculate the absolute number of hCD45+ cells (A), hCD3+ cells (B), T cell subpopulation (C) and subset of hCD4+ cells (D) and hCD8+ cells (E). Samples from spleen were analyzed by flow cytometry to calculate the absolute number of hCD19+ cells (F) and subtypes of hCD19+ cells (G). Data were analyzed 3 weeks after transplantation of hMNCs and treated with WT-MSCs or CXCR4-IL10-MSCs. Each bar represents the mean ± SEM. *p < 0.05; **p < 0.01. [file DataSheet_2.pdf]
